# Differences in characteristics of two aspects of procedural learning in action video game players

**DOI:** 10.7717/peerj.21013

**Published:** 2026-03-27

**Authors:** Wei Guo, Tao Tao, Jiahui Jiang, Biye Wang

**Affiliations:** College of Physical Education, Yangzhou University, Yangzhou, Jiangsu, China

**Keywords:** Action video games, Procedural learning, Statistical learning, Sequence learning

## Abstract

**Background:**

Action video game experience has been widely associated with differences in cognitive functions. However, it remains unclear whether such experience is differentially associated with distinct components of procedural learning, including statistical learning (sensitivity to probabilistic regularities) and higher-order sequence learning (extraction of serial order structures). This study aimed to examine descriptive patterns of these components using the Alternating Serial Reaction Time (ASRT) task.

**Methods:**

A total of 142 undergraduate students were recruited and categorized into action video game (AVG; *n* = 72) and non-video game (NVG; *n* = 70) group based on their self-reported gaming history. The ASRT task was used to evaluate procedural learning. Reaction times to three types of stimulus triplets were recorded across four epochs to assess general skill learning, statistical learning, and sequence learning. Mixed-design analysis of variance (ANOVAs) were conducted to examine learning effects and group differences.

**Results:**

AVG group exhibited significantly faster reaction times across all epochs, reflecting a descriptive pattern of faster general response speed. NVG group demonstrated a significant improvement in sensitivity to statistical regularities over time, whereas AVG group showed consistently lower levels of statistical learning. Conversely, AVG group showed reduced inverse learning effects in high-order sequence learning, suggesting better extraction of sequential structures.

**Conclusion:**

AVG experience was associated with faster motor responses and better sequence extraction ability, whereas NVG participants showed greater sensitivity to probabilistic regularities. These descriptive patterns are consistent with a multifactorial view of procedural learning and highlight potential interactions between statistical and sequence learning components.

## Introduction

A growing body of research suggests that action video game (AVG) training has been associated with enhanced general cognitive abilities, extending beyond game-specific skills to broader learning capabilities. In the field of education, numerous studies have demonstrated that video games improve students’ competencies in various domains, including mathematical reasoning (Dai et al., 2023; Nathan & Walkington, 2017), proportional mathematics (Graziano, Peterson & Shaw, 1999), and reading skills (Bavelier & Green, 2025; Pasqualotto et al., 2022). In medical education, proficiency in procedural skills such as laparoscopy (Gupta et al., 2021), bronchoscopy (Shimoda et al., 2024), endoscopy (Gugura et al., 2023; Hislop et al., 2006), and interventional radiology (Busch et al., 2021) has been positively correlated with video game experience (Lynch, Aughwane & Hammond, 2010). Similarly, within motor skill acquisition, video games, such as golf simulations, facilitate the development of force control in putting, translating virtual practice into real-world improvements (Fery & Ponserre, 2001; Pataky & Lamb, 2018). Therefore, the general learning-enhancement potential of action video games has been robustly established across various contexts.

Procedural learning, a fundamental cognitive ability essential for adapting to complex environmental stimuli, underpins numerous daily behaviors, including language acquisition, social interactions, and musical performance (Ashkenazi, Raiter-Avni & Vakil, 2022; Maie & Godfroid, 2025). Procedural learning involves recognizing and extracting both probability-based and sequential order-based regularities from environmental stimuli, mechanisms commonly termed statistical learning and higher-order sequence learning, respectively (Quentin et al., 2021; Simor et al., 2019; Zavecz et al., 2024). Such extracted information enables individuals to anticipate upcoming events and prepare suitable responses efficiently. Previous research indicates variability in procedural learning linked to individual experiences and conditions. For example, children with Tourette syndrome display enhanced sensitivity to statistical patterns but impaired sequential learning relative to typically developing peers (Tóth-Fáber et al., 2021b). Stress also differentially affects procedural learning, enhancing probability-based learning without altering sequence-based learning (Tóth-Fáber et al., 2021a). Age-related deficits have been reported, with younger individuals exhibiting superior internal knowledge acquisition compared to older adults (Ge et al., 2024). Moreover, expertise in specific skills, such as musical training, correlates positively with enhanced implicit sequence learning and faster reaction times to high-frequency sequences (Bergstrom, Howard & Howard, 2012). To our knowledge, few studies have explicitly demonstrated enhanced implicit regularity learning among experienced action video game players (AVGPs). Green, Pouget & Bavelier (2010) proposed that AVGPs exhibit superior probabilistic inference capabilities, utilizing stimulus evidence more efficiently, and further suggested that non-video game players (NVGPs) following intensive AVG training could experience enhanced sensitivity to probabilistic inference through gameplay.

Previous research established that AVG experience improves motor skill acquisition and enhances implicit probabilistic sequence learning (Bergstrom, Howard & Howard, 2012; Morin-Moncet et al., 2016). These studies indicate that AVGPs not only acquire domain-specific sequential relationships but also demonstrate heightened sensitivity to general probabilistic sequential patterns. Furthermore, recent findings suggest that video game expertise is linked to neural plasticity, manifesting in changes to brain connectivity that enhance cognitive functions such as attention and reasoning (Coronel-Oliveros et al., 2024). However, prior research has not rigorously differentiated between probability-based and order-based sequence learning. For instance, implicit probabilistic sequence learning has traditionally been measured through reaction time differences between high- and low-frequency triplets, conflating structured sequences with random ones. The Alternating Serial Reaction Time (ASRT) task effectively distinguishes these two aspects of procedural learning (Farkas et al., 2024; Simor et al., 2019). In this paradigm, participants rapidly respond to stimuli appearing sequentially in predefined locations, unbeknownst to participants, following a partially structured pattern. The alternating structure in ASRT tasks makes underlying sequences more difficult to detect explicitly compared to classic serial reaction time tasks. Additionally, ASRT has revealed phenomena such as reverse higher-order sequence learning, in which participants learn more slowly from deterministic sequences than from random sequences.

Potential cognitive mechanisms underlying the influence of video gaming on procedural learning include executive functions such as inhibition, set-shifting, and working memory. Studies have linked robust inhibitory control and cognitive flexibility to enhanced efficiency in statistical learning (Park et al., 2020). Given the well-documented positive impact of AVG on executive functions (Benoit et al., 2020; Bertoni et al., 2021; Wang, Jiang & Guo, 2023; Yu, Leung & Woo, 2021), a correlation between video game experience and statistical learning efficiency is plausible. However, findings remain inconsistent regarding the relationship between video gaming and procedural learning. For instance, research suggests that action video gamers exhibit increased overall visuomotor speed but no significant advantage in procedural motor learning, indicating a selective enhancement of visuomotor performance rather than procedural learning per se (Morin-Moncet et al., 2016).

Given these inconsistencies and superficial exploration in existing studies, the current research aims to investigate more thoroughly the relationships between different structural sequence types, probability variations, and AVG experience using the ASRT task.

## Materials and Methods

### Participants

Ninety-two undergraduate students were recruited through campus announcements and paper-based questionnaires assessing video game experience at Yangzhou University, China. Participants were categorized into two groups: action video game (AVG) and non-video game (NVG), based on self-reported gaming history from the past year using video game playing questionnaire (Foerster, Chidharom & Gierscha, 2023; Green et al., 2017). AVG participants were required to have engaged in at least five hours per week of video gaming over the past year, while NVG participants reported playing fewer than five hours per week during the same period. The games played by the AVG participants primarily included first-person shooters (*e.g.*, Call of Duty series, Overwatch, Counter Strike series), real-time strategy games (*e.g.*, Starcraft I, II & II, Dota), and multiplayer online battle arenas (*e.g.*, League of Legends, Heroes of the Storm), which combine characteristics of action and real-time strategy games.

Following the reviewers’ suggestion to increase statistical power, an additional round of participant recruitment was carried out after the initial data collection, resulting in two recruitment rounds in total. The first round recruited 92 students, and the second round recruited an additional 60 students using the same inclusion criteria and identical experimental procedures. Across both rounds, two participants failed to complete the experiment, and eight participants were excluded due to low accuracy (below 80%). The demographic characteristics (age and gender distribution) of participants in the two recruitment rounds were comparable, and no significant between-round differences were observed. In total, 142 participants were included in the final dataset. The AVG group consisted of 72 participants (40 males and 32 females; *M* age = 19.15 years, *SD* age = 0.62 years), with weekly gaming hours ranging from 15 to 38. NVG group included 70 participants (32 males and 38 females; *M* age = 19.05 years, *SD* age = 0.58 years), with weekly gaming hours ranging from 0 to 4.

All participants reported normal or corrected-to-normal vision and no history of neurological, psychiatric, or chronic medical conditions. Prior to participation, written informed consent was obtained from all participants. This study was approved by the Ethics Committee of Yangzhou University (No.YXYLL-2023-083) and carried out in compliance with the approved guidelines. Participants in the current cross-sectional study were explicitly informed that the study aimed to explore aspects of cognitive processing but were not informed about the specific nature of the video-game-related hypotheses or expected group differences (Denkinger et al., 2021).

### The Alternating Serial Reaction Time (ASRT) task

**Figure 1:**
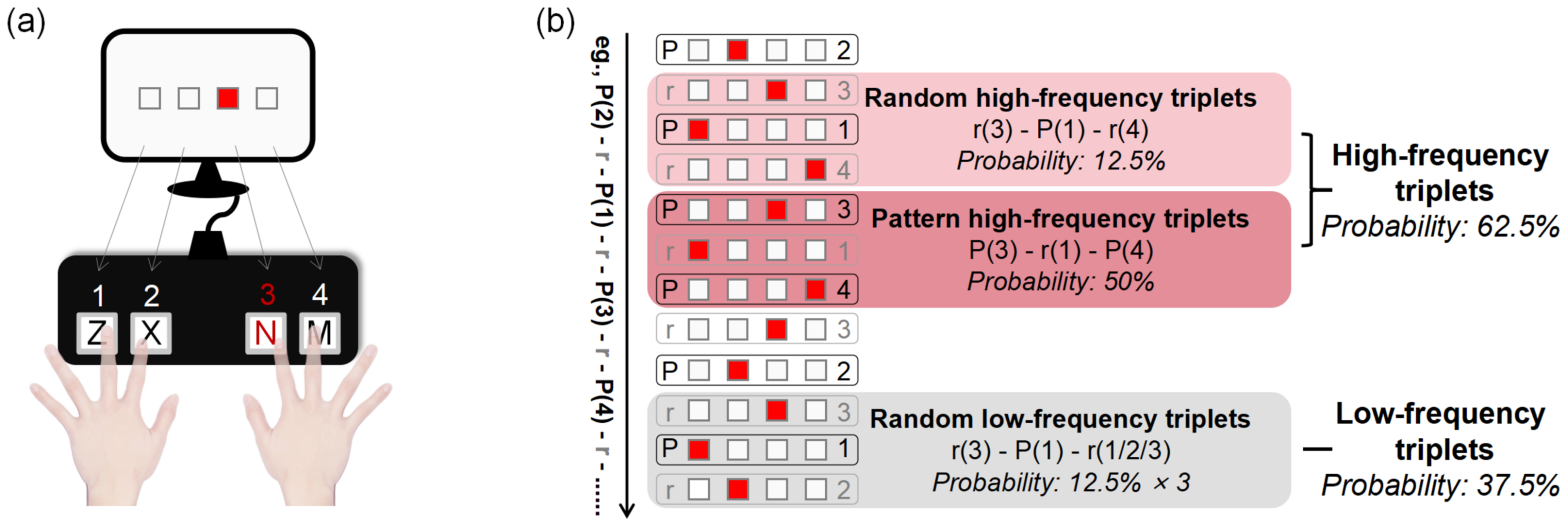
Diagram of the experimental task. (A) Alternating Serial Reaction Time (ASRT) task. (B) Schematic representation of triplet structures and their corresponding occurrence probabilities in the ASRT task.

During the ASRT task (Hunt & Aslin, 2001; Si et al., 2022), four empty squares were horizontally arranged on a gray screen. On each trial, one square was filled with red, and participants pressed the corresponding key (Z, X, N, or M) on a standard keyboard using the left and right index and middle fingers (Fig. 1A). The stimulus remained visible until the correct response was made, followed by a 120 ms inter-stimulus interval. Each participant completed 20 blocks of 85 trials. The first five trials in each block were random practice trials and were excluded from analysis.

The presentation of the stimuli followed an 8-element alternating sequence in which fixed (pattern) and random elements alternated (*e.g.*, 2–r–1–r–3–r–4–r, where numbers refer to fixed locations and “r” denotes a randomly selected location). Six possible sequence permutations were used (*e.g.*, 2–r–1–r–3–r–4–r or 2–r–4–r–3–r–1–r), and one sequence was randomly assigned to each participant, counterbalanced across groups.

This sequence design generates three types of triplets, sets of three successive elements that differ in their frequency of occurrence.

High-frequency triplets (*e.g.*, 2–X–1, 1–X–3, 3–X–4, 4–X–2, where X is the middle element) appear often because their first and last elements correspond to two adjacent pattern elements. These may occur either as pattern high-frequency triplets (50% probability) or random high-frequency triplets (12.5% probability), together forming a total occurrence probability of 62.5%.

In contrast, random low-frequency triplets (*e.g.*, 2–X–3 or 1–X–4) can only occur when both the first and last elements are random, yielding a total probability of 37.5% (12.5% ×3) (Fig. 1B). All schematic illustrations presented in Fig. 1 were independently created by the authors for the present study.

Based on these triplet types, three learning components were assessed: (1) General skill learning, reflected by overall reductions in reaction times (RTs) across epochs; (2) statistical learning, reflected by faster RTs to random high- than random low-frequency triplets; and (3) sequence learning, reflected by faster RTs to pattern high- than random high-frequency triplets. These distinctions allow the ASRT task to separately evaluate probabilistic and sequential aspects of procedural learning.

### Procedure

Eligible participants performed the ASRT task separately in a quiet, dimly lit room after fully understanding the task instructions. Each participant sat comfortably about 60 cm in front of a computer screen. Instructions were displayed on the screen to guide participants to start the experimental task by pressing the space bar on the keyboard. During the task, participants were instructed to respond as quickly and accurately as possible to visual stimuli by pressing the corresponding keys on a QWERTY keyboard: “Z” with the left middle finger, “X” with the left index finger, “N” with the right index finger, and “M” with the right middle finger. The ASRT task consisted of 20 blocks in total. Upon completion of each block, participants were provided with feedback displaying their average response time and accuracy for that block. A mandatory rest period of at least 20 s was given before the start of the next block. The entire task lasted approximately 20 min.

### Statistical analysis

The 20 blocks of the ASRT task were averaged into four epochs to capture the progression of learning over time. To control for pre-existing response tendencies, repetitions (*e.g.*, 111) and trills (*e.g.*, 121), as well as trials with incorrect responses, were excluded. Additionally, trials with incorrect responses were removed. For each participant, the median reaction time (RT) of correct responses was calculated separately for each epoch. Each trial was categorized as the third element in a pattern-high, random-high, or random-low triplet.

General Learning was operationalized as a reduction in median RT across successive epochs, reflecting improvement over time. A 4 (epoch) ×2 (group) mixed-design analysis of variance (ANOVA) was conducted on median RTs, with epoch as the within-subjects factor and group as the between-subjects factor.

Statistical learning was defined as faster responses to random high-frequency triplets compared to random low-frequency triplets. A 4 (epoch) ×2 (frequency: high *vs.* low) ×2 (group) mixed-design ANOVA was conducted on the median RTs of the random triplets. Epoch and frequency were within-subject factors, and group was a between-subjects factor. The statistical learning effect was computed by subtracting the median RT of random high-frequency triplets from that of random low-frequency triplets. A separate 4 × 2 mixed-design ANOVA was conducted on this effect, with epoch as the within-subjects factor and group as the between-subjects factor.

Sequence learning was defined as faster responses to pattern high-frequency triplets relative to random high-frequency triplets. A 4 (epoch) ×2 (frequency: pattern high *vs.* random high) ×2 (group) mixed-design ANOVA was used to analyze the corresponding median RTs. Epoch and frequency were within-subject factors, and group was the between-subjects factor. The sequence learning effect was determined by subtracting the median RT of pattern high-frequency triplets from that of random high-frequency triplets. This learning effect was further analyzed using a 4 × 2 mixed-design ANOVA with epoch and group as factors.

**Table 1:** Mean response time and standard deviation for the four epochs.

**Epoch**	**AVG group**				**NVG group**			
	**Overall**	**Pattern high-frequency**	**Random high-frequency**	**Random low-frequency**	**Overall**	**Pattern high-frequency**	**Random high-frequency**	**Random low-frequency**
1	370.27 ± 43.50	370.93 ± 42.65	365.90 ± 44.11	373.79 ± 47.52	407.65 ± 52.36	406.96 ± 54.88	404.62 ± 47.98	411.84 ± 53.32
2	372.85 ± 42.47	369.51 ± 44.98	369.83 ± 41.98	379.01 ± 45.61	410.65 ± 52.20	406.08 ± 53.57	404.86 ± 51.09	417.67 ± 54.62
3	361.13 ± 39.00	361.02 ± 40.56	356.26 ± 40.18	364.95 ± 41.94	401.29 ± 45.47	398.31 ± 46.22	394.57 ± 45.37	410.85 ± 48.96
4	356.00 ± 35.52	352.38 ± 37.57	354.28 ± 38.17	363.42 ± 38.27	390.50 ± 41.07	387.72 ± 43.30	378.29 ± 40.11	401.06 ± 41.53

## Results

### General skill learning

General skill learning was assessed by analyzing RTs across epochs in the AVG and NVG groups. A mixed ANOVA revealed a significant main effect of epoch (F(2.10, 293.89) = 40.44, *p* < 0.001, *η*_*p*_^2^ = 0.224), showing a decrease in RTs as the task progressed. *Post-hoc* comparisons confirmed a consistent decrease in RTs across epochs in both groups, with performance improving steadily from epoch 1 to epoch 4 (all *ps* < 0.01). A significant main effect of group (F(1, 140) = 28.06, *p* < 0.001, *η*_*p*_^2^ = 0.167) revealed faster RTs in the AVG group (*M* = 365.06 ms, *SD* = 38.17) compared to the NVG group (*M* = 402.52 ms, *SD* = 45.85). No significant interaction between epoch and group was found (F(2.10, 293.89) = 0.79, *p* = 0.460, *η*_*p*_^2^ = 0.006).

### Statistical learning

Table 1 presents the mean RTs for random high- and low-frequency triplets across epochs for both groups. Descriptively, RTs decreased over time, indicating practice-related improvement, and the AVG group responded faster overall than the NVG.

**Figure 2:**
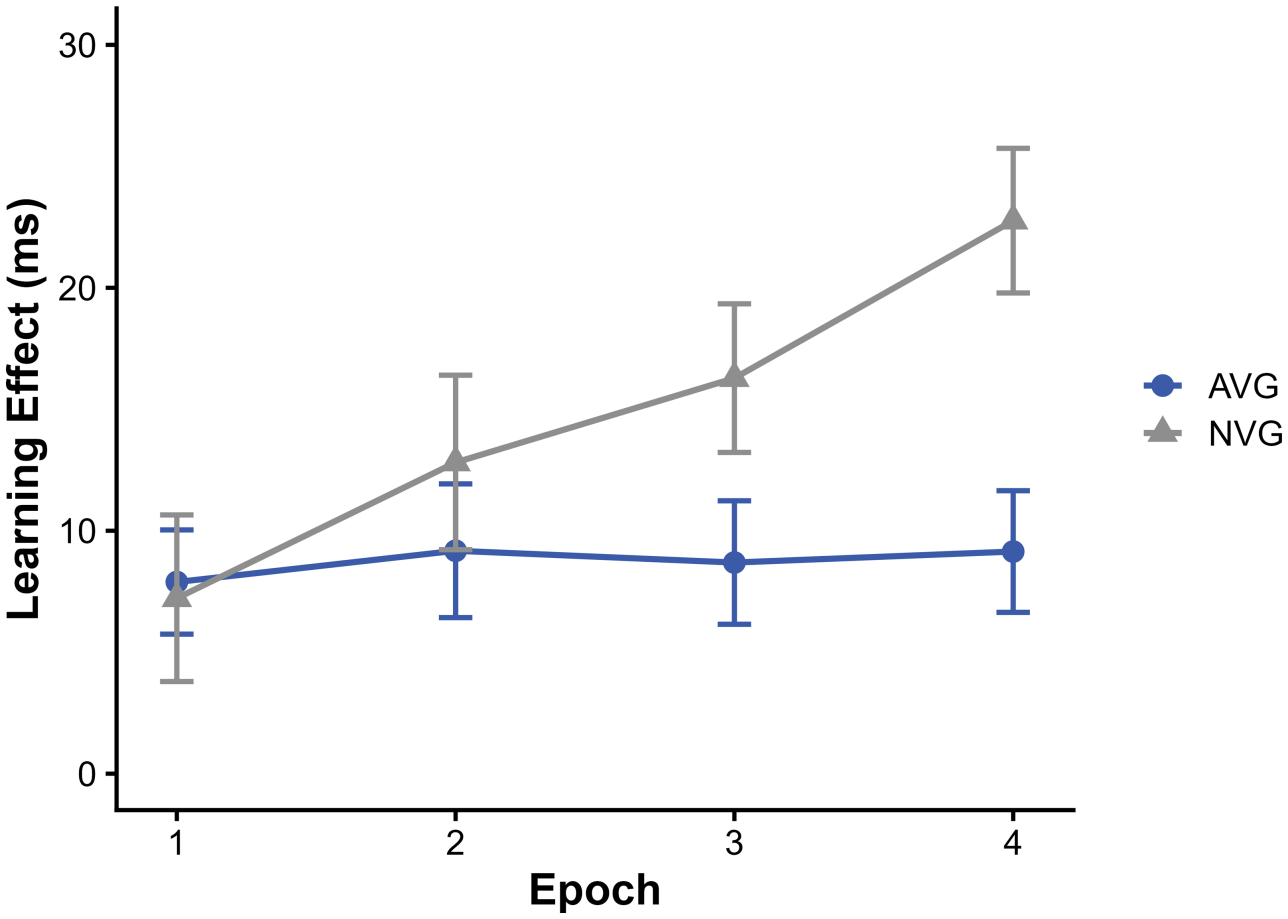
Statistical learning effects in four epochs.

To isolate statistical learning, we computed the learning effect as the RT difference between random low- and random high-frequency triplets (Fig. 2). A mixed ANOVA revealed significant main effects of group (F(1, 140) = 5.60, *p* = 0.019, *η*_*p*_^2^ = 0.015) and epoch (F(2.81, 393.85) = 3.53, *p* = 0.017, *η*_*p*_^2^ = 0.015). In addition, a statistically significant group × epoch interaction was found (F (2.81, 393.85) = 2.69, *p* = 0.048, *η*_*p*_^2^ = 0.012), although the associated effect size was small.

Exploratory group comparisons within each epoch showed no reliable group differences in epochs 1 or 2 (*ps* > 0.42). A marginal difference was observed in epoch 3 (*p* = 0.057), and a group difference was observed in epoch 4, with the NVG group showing a larger numerical statistical learning effect than the AVG group (*p* = 0.0006). Given the absence of a robust main effect of group across epochs and the small interaction effect size, these epoch-specific contrasts are reported descriptively. Within-group analyses indicated that the NVG group showed an increase in statistical learning across practice, with epoch 4 differing from epoch 1 (*p* = 0.0004) and epoch 2 (*p* = 0.0247). In contrast, no reliable change in the learning effect across epochs was observed in the AVG group (all *ps* > 0.99 after Bonferroni correction). Taken together, these findings describe different learning trajectories across practice between groups. However, given the small effect sizes, these patterns should be interpreted as descriptive rather than as evidence of robust group differences in statistical learning.

### High-order sequence learning

Table 1 includes the mean RTs for the triplet types relevant to high-order sequence learning (pattern high-frequency and random high-frequency), arranged across epochs for both groups. Overall, RTs decreased with practice, and AVG participants responded faster than NVG participants.

**Figure 3:**
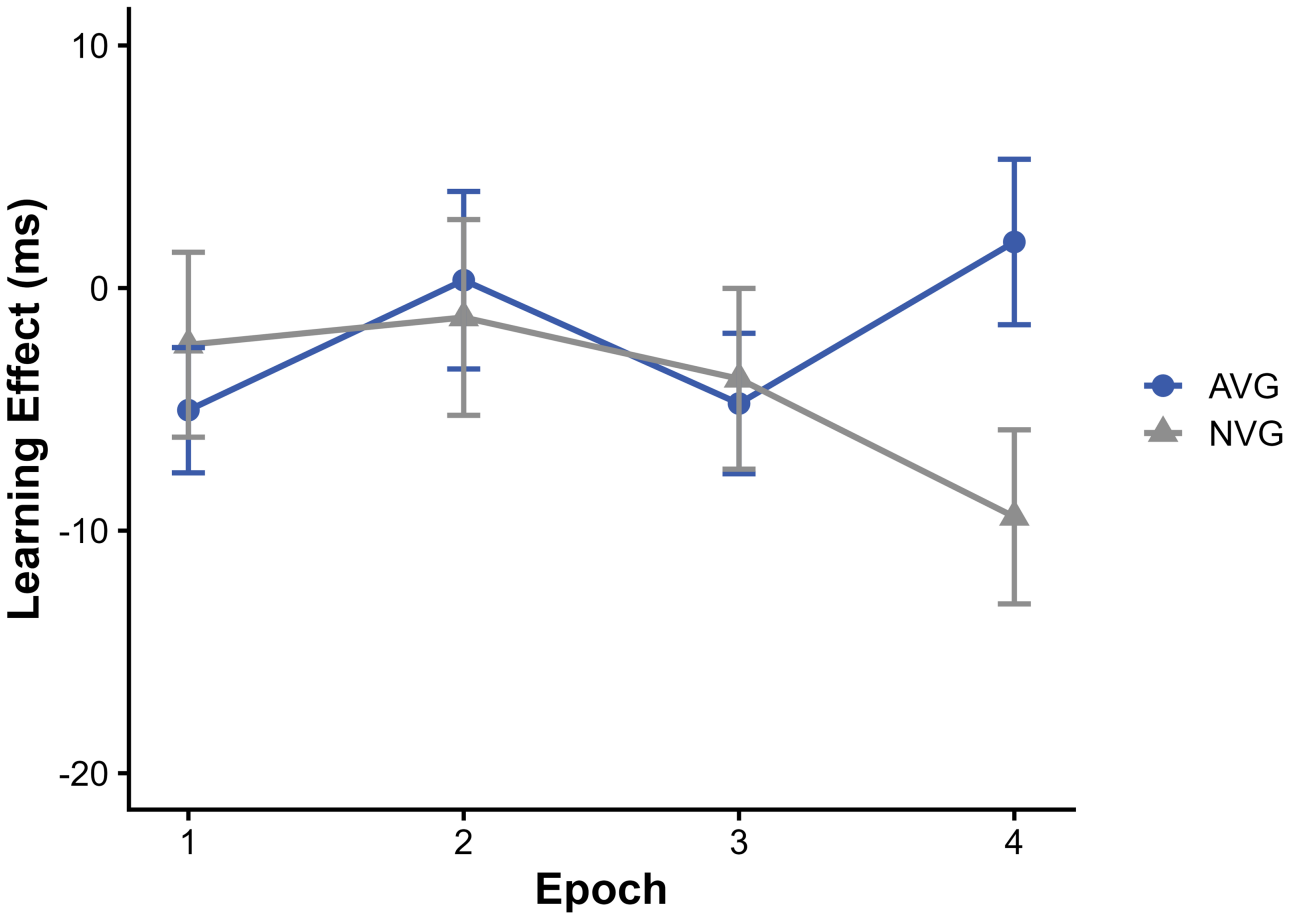
High-order sequence learning effects in four epochs.

To examine high-order sequence learning, we computed the sequence learning effect as the RT difference between random high- and pattern high-frequency triplets (Fig. 3). A mixed ANOVA revealed no significant main effect of group, F(1, 140) = 0.32, *p* = 0.571, *η*_p_^2^ =0.002, and no significant main effect of epoch, F(2.88, 402.60) = 1.13, *p* = 0.337, *η*_p_^2^ = 0.003. A statistically significant group × epoch interaction was observed, F(2.88, 402.60) = 3.64, *p* = 0.014, *η*_p_^2^ = 0.009, although the associated effect size was small. Exploratory simple effects analyses were conducted to describe this interaction. Group comparisons within each epoch revealed no significant differences between AVG and NVG in epochs 1–3 (all *ps* > 0.55). A group difference emerged in epoch 4, where the NVG group showed a more negative learning effect than the AVG group (*p* = 0.024). Given the absence of significant main effects and the small interaction effect size, this late-epoch difference is reported descriptively.

For completeness, the full results of the accuracy analyses and the detailed ANOVA tables for all RT data (including all main and interaction effects) are provided in the Supplementary File.

## Discussion

The present study aimed to investigate whether and how experience with AVG is associated with differences in two aspects of procedural learning: statistical learning and high-order sequence learning. We employed a non-prompted version of the ASRT task, which allowed us to simultaneously assess and clearly distinguish these two procedural learning components within a single experimental paradigm. Our findings indicate that video game experience was associated with faster RTs, whereas differences in statistical learning and high-order sequence learning were reflected in distinct descriptive learning patterns across practice.

These results are consistent with previous research showing that video game experience is associated with faster reaction speed (Dye, Green & Bavelier, 2009; Güney et al., 2024). However, contrary to earlier findings suggesting that video gaming facilitates the extraction of probabilistic knowledge (Morin-Moncet et al., 2016), the present results descriptively showed that the NVG group exhibited a progressive increase in statistical learning across epochs, whereas the AVG group displayed a relatively stable level of probabilistic sensitivity across practice. A statistically significant group-by-epoch interaction was observed in statistical learning. However, given the small effect size and the absence of a robust main effect of group, this interaction is interpreted as reflecting descriptive differences in learning trajectories rather than robust group differences. NVG participants showed a increase in statistical learning from early to later epochs, whereas the AVG group exhibited little change across the task. Accordingly, the present findings suggest that AVG experience may be associated with a more limited degree of late-stage improvement in statistical learning, rather than with an absence of statistical learning per se. In this context, it is worth noting that Bergstrom, Howard & Howard (2012) assessed learning using a single learning score contrasting low- and high-frequency triplets. When the same learning score is applied here, the group difference does not reach significance, suggesting that examining statistical learning and high-order sequence learning separately may be more sensitive to group differences than using this learning score alone.

One possible interpretation of the observed group differences in statistical learning across epochs may relates to the interplay between executive functions and procedural learning (Park et al., 2020). Learning is typically considered complete when it becomes automatized, requiring minimal attentional and inhibitory resources. Early stages of learning demand high levels of attentional and inhibitory control, whereas later stages involve greater automaticity and reduced executive involvement. Previous studies have shown that a reduction in attentional and inhibitory control can be associated with enhanced statistical learning and increased sensitivity to probabilistic regularities. In contrast, AVGPs have consistently demonstrated superior attentional and inhibitory capacities. From this perspective, the descriptive pattern observed in the present study may reflect differences in learning dynamics associated with executive efficiency, rather than indicating a definitive mechanistic explanation. Specifically, the relatively lower statistical learning sensitivity observed in AVGPs may be interpreted as a potential consequence of earlier automatization or reduced need for further adjustment during later practice stages, rather than as evidence of impaired learning.

The ASRT task has two widely used variants: the cued version, where deterministic and random trials are visually distinct, and the un-cued version, where they are indistinguishable. We employed the un-cued ASRT in this study, as it is particularly suited for examining implicit learning without the aid of external cues. In relatively short sessions, learning in the un-cued ASRT paradigm may exhibit an inverse sequence learning effect, sometimes referred to as a negative learning effect, whereby responses to random-high triplets are faster than to pattern-high triplets (Song, Howard & Howard, 2007). A similar response pattern was also observed in the present study. Recent computational accounts, such as the hierarchical Bayesian sequence model of Élteto et al. (2022), suggest that such negative learning effects may emerge because learners initially rely on local statistical fluctuations (*e.g.*, trigram recency) before gradually stabilizing higher-order sequential representations. Within this framework, the reduced inverse sequence learning observed in AVGPs may descriptively reflect differences in how participants transition from reliance on local statistics toward the extraction of more global sequential information.

Considering that statistical and sequential learning showed opposite trajectories in our participants, our findings are consistent with the view that these two forms of learning (frequency-based *vs.* order-based processing) are at least partly distinct (Tóth-Fáber et al., 2021a; Tóth-Fáber et al., 2021b). Previous studies indicate that statistical learning typically occurs rapidly and incidentally, whereas sequence learning develops more gradually through repeated exposure, irrespective of conscious intent (Simor et al., 2019). Neurophysiological evidence from event-related potentials and oscillatory measures further supports their partial independence at the neural level (Kóbor et al., 2018). The current study aligns with this multifactorial perspective of procedural learning and suggests, at a descriptive level, the possibility of dynamic interactions between statistical and sequential learning components (Tóth-Fáber et al., 2021a; Tóth-Fáber et al., 2021b).

Several limitations should be acknowledged. First, our analyses were primarily phenomenological and based on a cross-sectional, case-control design, which does not allow us to determine whether the observed differences reflect descriptive associations with video game experience itself or are attributable to pre-existing individual characteristics that might facilitate gaming performance. Second, gameplay time was used only as a basic inclusion criterion rather than a continuous measure, which prevented examination of how differences in gaming intensity or history might contribute to individual variability. Third, this study did not further distinguish between different types of AVGs, as the relatively small sample size limited the feasibility of such subdivision and might have further constrained statistical power for detecting subtle effects. Finally, the analyses relied on traditional frequentist methods, which restrict the ability to quantify the strength of evidence for or against the observed effects, particularly in the context of small effect sizes and interaction-based findings.

Future studies should address these limitations by integrating neural measures, obtaining more fine-grained and continuous assessments of gaming experience, and systematically comparing specific types of video games, in order to more precisely characterize how gaming is associated with procedural learning. In addition, the application of Bayesian approaches may help quantify the strength of evidence more effectively and thereby improve the robustness and interpretability of future findings.

## Conclusion

In summary, the present study shows that action video game experience is associated with faster reaction speed. Specifically, action video game players exhibited faster overall responses and relatively better sequence extraction ability, whereas non-video game players showed more pronounced practice-related increases in sensitivity to statistical regularities. These dissociable patterns underscore the multifaceted nature of procedural learning and suggest that different components of procedural learning may be differentially shaped by experience. Future longitudinal and neurocognitive research will be important for clarifying the mechanisms underlying these trajectory differences.

##  Supplemental Information

10.7717/peerj.21013/supp-1 Supplemental Information 1 Raw data for reaction time

10.7717/peerj.21013/supp-2 Supplemental Information 2 Raw data for accuracy

10.7717/peerj.21013/supp-3 Supplemental Information 3 RH vs RL RT

10.7717/peerj.21013/supp-4 Supplemental Information 4 PH vs RH RT

10.7717/peerj.21013/supp-5 Supplemental Information 5 ACC with ANOVA

10.7717/peerj.21013/supp-6 Supplemental Information 6 SL HSL Effect ANOVAs

## Additional Information and Declarations

### Data Availability

The following information was supplied regarding data availability: The raw data are available in the Supplemental Files.
