# Supplementary figures and images for: Differences in characteristics of two aspects of procedural learning in action video game players
Source: PeerJ. 2026 Mar 27;14:e21013. doi: 10.7717/peerj.21013 (PMC13034865; doi:10.7717/peerj.21013)

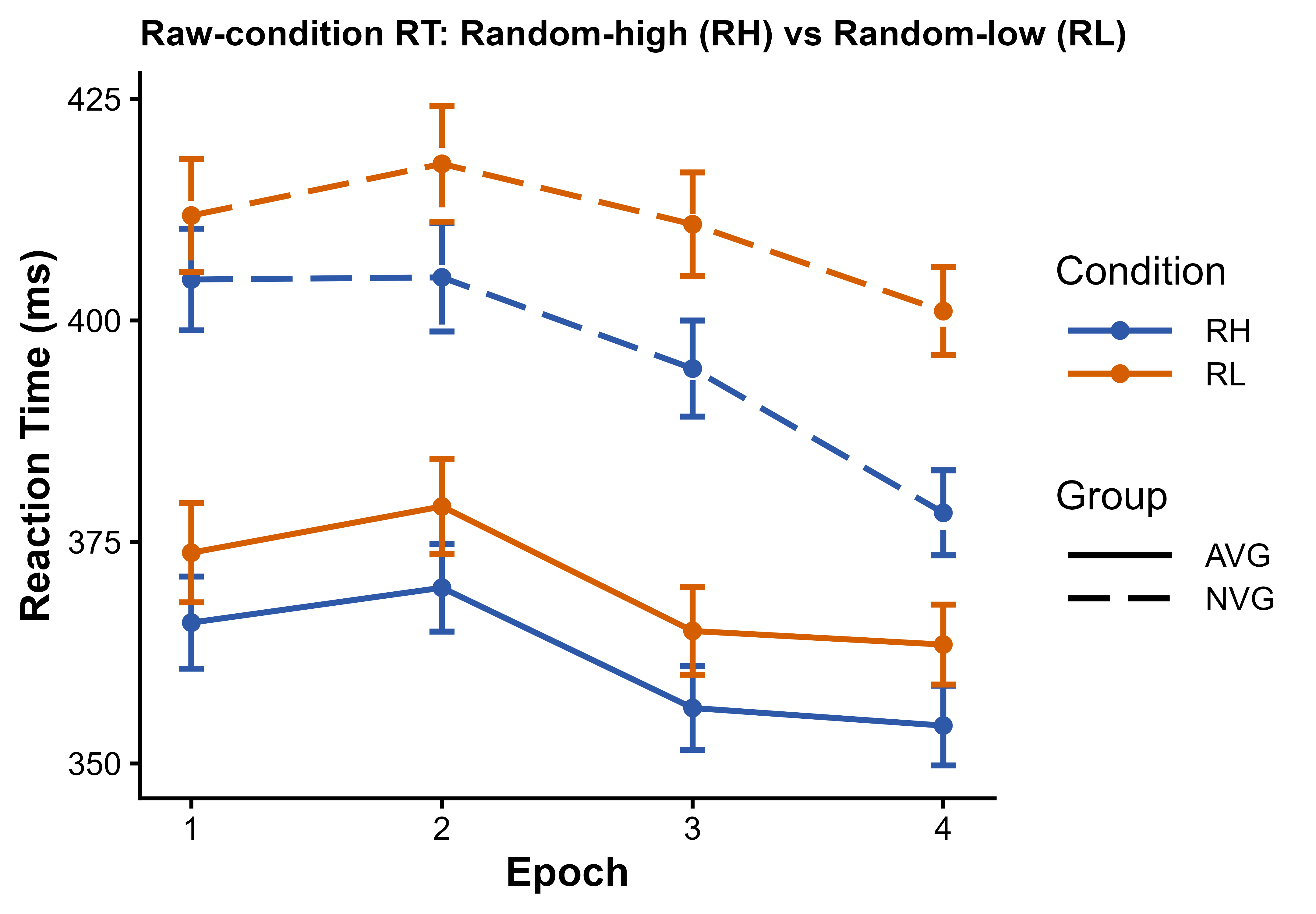

Supplement: Supplemental Information 3 [file peerj-14-21013-s003.png]

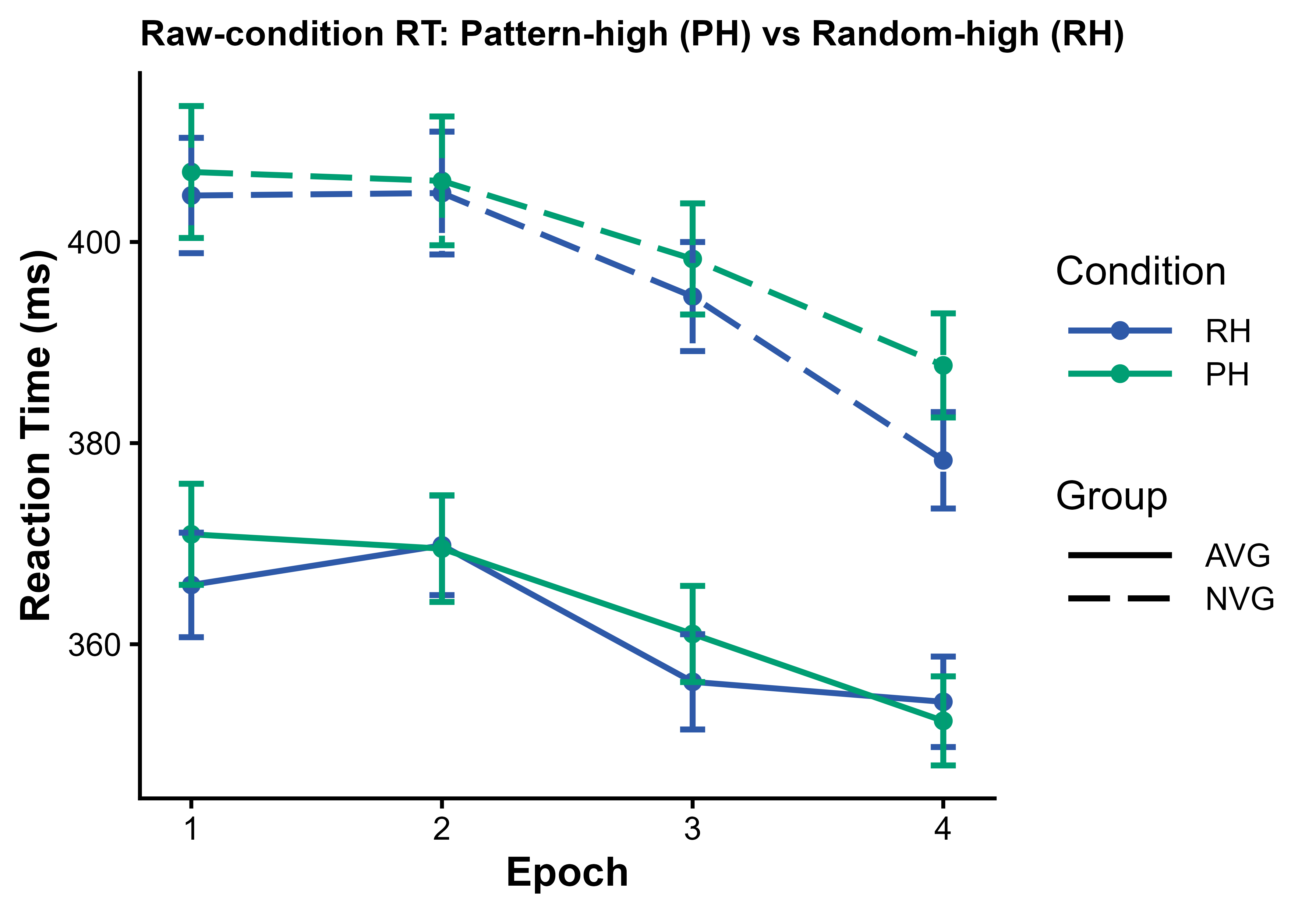

Supplement: Supplemental Information 4 [file peerj-14-21013-s004.png]
