# Supplementary material for: Differences in characteristics of two aspects of procedural learning in action video game players
Source: PeerJ. 2026 Mar 27;14:e21013. doi: 10.7717/peerj.21013 (PMC13034865; doi:10.7717/peerj.21013)
Supplement: Supplemental Information 5 [file peerj-14-21013-s005.docx]

Table S1. Mean accuracy (± SD) across the four epochs for the AVG and NVG groups.

| **Epoch** | **AVG Group** | **NVG Group** |
| --- | --- | --- |
| 1 | 0.96±0.02 | 0.95±0.03 |
| 2 | 0.94±0.03 | 0.94±0.03 |
| 3 | 0.93±0.04 | 0.93±0.04 |
| 4 | 0.93±0.05 | 0.92±0.04 |

## Statistical Analysis of Accuracy (Overall ACC)

A mixed-design ANOVA with Group (AVG vs. NVG) as a between-subject factor and Epoch (1–4) as a within-subject factor was conducted on overall accuracy.

### ANOVA Table

| **Effect** | **num Df** | **den Df** | **MSE** | **F** | **ηp²** | **Pr(>F)** |
| --- | --- | --- | --- | --- | --- | --- |
| group | 1.000000 | 140.0000 | 0.003692808 | 0.50 | 0.004 | 0.481 |
| epoch | 2.265646 | 317.1905 | 0.000751749 | 45.72 | 0.246 | < .001 |
| group:epoch | 2.265646 | 317.1905 | 0.000751749 | 0.20 | 0.001 | 0.842 |

### Simple Effects: Group within Each Epoch (Bonferroni)

| **contrast** | **epoch** | **estimate** | **SE** | **df** | **t.ratio** | **p.value** |
| --- | --- | --- | --- | --- | --- | --- |
| AVG - NVG | X1 | 0.005 | 0.004 | 140 | 1.27 | 0.206 |
| AVG - NVG | X2 | 0.003 | 0.005 | 140 | 0.51 | 0.609 |
| AVG - NVG | X3 | 0.001 | 0.007 | 140 | 0.21 | 0.836 |
| AVG - NVG | X4 | 0.005 | 0.008 | 140 | 0.68 | 0.498 |

### Simple Effects: Epoch within Each Group (Bonferroni)

| **contrast** | **group** | **estimate** | **SE** | **df** | **t.ratio** | **p.value** |
| --- | --- | --- | --- | --- | --- | --- |
| X1 - X2 | AVG | 0.021 | 0.003 | 140 | 6.82 | < .001 |
| X1 - X3 | AVG | 0.029 | 0.004 | 140 | 6.38 | < .001 |
| X1 - X4 | AVG | 0.030 | 0.005 | 140 | 6.24 | < .001 |
| X2 - X3 | AVG | 0.008 | 0.004 | 140 | 2.12 | 0.216 |
| X2 - X4 | AVG | 0.010 | 0.004 | 140 | 2.37 | 0.114 |
| X3 - X4 | AVG | 0.001 | 0.003 | 140 | 0.43 | 1.000 |
| X1 - X2 | NVG | 0.018 | 0.003 | 140 | 5.98 | < .001 |
| X1 - X3 | NVG | 0.025 | 0.005 | 140 | 5.51 | < .001 |
| X1 - X4 | NVG | 0.030 | 0.005 | 140 | 6.18 | < .001 |
| X2 - X3 | NVG | 0.007 | 0.004 | 140 | 1.76 | 0.489 |
| X2 - X4 | NVG | 0.012 | 0.004 | 140 | 2.93 | 0.024 |
| X3 - X4 | NVG | 0.005 | 0.003 | 140 | 1.51 | 0.794 |

## Accuracy by Condition (RH, RL, PH)

Mean accuracy (± SD) for random high-frequency (RH), random low-frequency (RL), and pattern high-frequency (PH) trials across epochs.

| **Epoch** | **AVG_PH** | **AVG_RH** | **AVG_RL** | **NVG_PH** | **NVG_RH** | **NVG_RL** |
| --- | --- | --- | --- | --- | --- | --- |
| 1 | 0.96±0.02 | 0.96±0.02 | 0.96±0.03 | 0.96±0.03 | 0.96±0.03 | 0.95±0.03 |
| 2 | 0.94±0.04 | 0.94±0.03 | 0.94±0.04 | 0.94±0.03 | 0.94±0.03 | 0.93±0.04 |
| 3 | 0.93±0.04 | 0.93±0.04 | 0.93±0.05 | 0.93±0.04 | 0.93±0.04 | 0.93±0.06 |
| 4 | 0.93±0.05 | 0.93±0.05 | 0.92±0.05 | 0.93±0.04 | 0.93±0.04 | 0.92±0.05 |

### Mixed-design ANOVA (Group × Condition × Epoch)

| **Effect** | **num Df** | **den Df** | **MSE** | **F** | **ηp²** | **p** |
| --- | --- | --- | --- | --- | --- | --- |
| group | 1.000000 | 140.0000 | 0.0110832891 | 0.58 | 0.004 | 0.448 |
| Condition | 1.108153 | 155.1414 | 0.0007023202 | 26.43 | 0.159 | < .001 |
| group:Condition | 1.108153 | 155.1414 | 0.0007023202 | 0.95 | 0.007 | 0.341 |
| Epoch | 2.274594 | 318.4431 | 0.0022756873 | 45.09 | 0.244 | < .001 |
| group:Epoch | 2.274594 | 318.4431 | 0.0022756873 | 0.17 | 0.001 | 0.871 |
| Condition:Epoch | 3.249851 | 454.9791 | 0.0006175072 | 0.96 | 0.007 | 0.414 |
| group:Condition:Epoch | 3.249851 | 454.9791 | 0.0006175072 | 1.21 | 0.009 | 0.306 |

## Accuracy by Condition (RH, RL, PH)

Mean accuracy (± SD) for random high-frequency (RH), random low-frequency (RL), and pattern high-frequency (PH) trials across epochs.

| **Epoch** | **AVG_PH** | **AVG_RH** | **AVG_RL** | **NVG_PH** | **NVG_RH** | **NVG_RL** |
| --- | --- | --- | --- | --- | --- | --- |
| 1 | 0.96±0.02 | 0.96±0.02 | 0.96±0.03 | 0.96±0.03 | 0.96±0.03 | 0.95±0.03 |
| 2 | 0.94±0.04 | 0.94±0.03 | 0.94±0.04 | 0.94±0.03 | 0.94±0.03 | 0.93±0.04 |
| 3 | 0.93±0.04 | 0.93±0.04 | 0.93±0.05 | 0.93±0.04 | 0.93±0.04 | 0.93±0.06 |
| 4 | 0.93±0.05 | 0.93±0.05 | 0.92±0.05 | 0.93±0.04 | 0.93±0.04 | 0.92±0.05 |

### Mixed-design ANOVA (Group × Condition × Epoch)

| **Effect** | **num Df** | **den Df** | **MSE** | **F** | **ηp²** | **p** |
| --- | --- | --- | --- | --- | --- | --- |
| group | 1.000000 | 140.0000 | 0.0110832891 | 0.58 | 0.004 | 0.448 |
| Condition | 1.108153 | 155.1414 | 0.0007023202 | 26.43 | 0.159 | < .001 |
| group:Condition | 1.108153 | 155.1414 | 0.0007023202 | 0.95 | 0.007 | 0.341 |
| Epoch | 2.274594 | 318.4431 | 0.0022756873 | 45.09 | 0.244 | < .001 |
| group:Epoch | 2.274594 | 318.4431 | 0.0022756873 | 0.17 | 0.001 | 0.871 |
| Condition:Epoch | 3.249851 | 454.9791 | 0.0006175072 | 0.96 | 0.007 | 0.414 |
| group:Condition:Epoch | 3.249851 | 454.9791 | 0.0006175072 | 1.21 | 0.009 | 0.306 |
